# On the Effect of Nanoparticle Surface Chemistry on the Electrical Characteristics of Epoxy-Based Nanocomposites

**DOI:** 10.3390/polym8040126

**Published:** 2016-04-06

**Authors:** Celia Yeung, Alun S. Vaughan

**Affiliations:** Department of Electronics and Computer Science, University of Southampton, Southampton SO17 1BJ, UK; miss.c.yeung@googlemail.com

**Keywords:** nanoparticle, surface functionalization, nanocomposite, morphology, dielectric breakdown strength, dielectric relaxation

## Abstract

The effect of nanosilica surface chemistry on the electrical behavior of epoxy-based nanocomposites is described. The nanosilica was reacted with different volumes of (3-glycidyloxypropyl)trimethoxysilane and the efficacy of the process was demonstrated by infrared spectroscopy and combustion analysis. Nanocomposites containing 2 wt % of nanosilica were prepared and characterized by scanning electron microscopy (SEM), AC ramp electrical breakdown testing, differential scanning calorimetry (DSC) and dielectric spectroscopy. SEM examination indicated that, although the nanoparticle dispersion improved somewhat as the degree of surface functionalization increased, all samples nevertheless contained agglomerates. Despite the non-ideal nature of the samples, major improvements in breakdown strength (from 182 ± 5 kV·mm^−1^ to 268 ± 12 kV·mm^−1^) were observed in systems formulated from optimally treated nanosilicas. DSC studies of the glass transition revealed no evidence for any modified interphase regions between the nanosilica and the matrix, but interfacial effects were evident in the dielectric spectra. In particular, changes in the magnitude of the real part of the permittivity and variations in the interfacial α′-relaxation suggest that the observed changes in breakdown performance stem from variations in the polar character of the nanosilica surface, which may affect the local density of trapping states and, thereby, charge transport dynamics.

## 1. Introduction

Composites have long been used in a wide range of different applications since, by combining two or more components, it is possible to generate material systems with improved properties. Nevertheless, the need for new materials with increased functionality has led to increasing interest in a new generation of composite materials, namely nanocomposites, since these have the potential to deliver combinations of properties that are not accessible through other routes [1,2]. These filled systems differ from conventional composite materials as the filling phase measures a maximum of 100 nm in at least one dimension and, consequently, the specific interfacial area between the matrix and the filler is large. Indeed, it has been suggested that many aspects of nanocomposite behavior are dominated by interfacial effects related to the presence of interphases, which constitute a significant volume fraction of the system [3].

Although considerable initial effort was focused on generating nanocomposites with tailored thermomechanical properties, the concept of “nanodielectrics” was introduced by Lewis in 1994 [3] and quickly gathered momentum in the field of electrical insulation. As the demands on electrical power equipment grow, so do those imposed on their insulation, and may include: improved electrical breakdown strength, reduced dielectric loss, increased thermal conductivity, modified charge transport dynamics, *etc*. In the case of nanodielectrics, many beneficial effects have been attributed to the introduction of nanoparticles [4,5,6], but, as a consequence of their nanometric size, nanofillers tend to aggregate prior to and during nanocomposite production, which can impair properties. In particular, the use of polar fillers in non-polar dielectric media can be problematical, as typical mixing techniques are in constant competition with high surface energies and electrostatic forces that can adversely affect the homogeneity of the nanofiller dispersion within the matrix.

To address such compatibility issues, many studies have employed the use of coupling agents, where organofunctional surface reactants are used to modify the mineral substrate surface in order to improve interactions between the nanofiller and the matrix polymer. One of the most commonly employed coupling strategies exploits organosilanes and a number of publications have shown how the use of such compounds can lead to improved filler dispersion in nanodielectrics, thereby enhancing the resulting electrical properties of the system [7,8]. Determining the optimum quantity of coupling agent for functionalization is, however, of great importance. If insufficient coupling agent is used, the interactions at the nanofiller-matrix interface will not be optimal and weaker matrix bonding may result while, if excess coupling agent is used, self-condensation may prove disadvantageous [7]. Methods for estimating the optimum quantity of coupling agent have therefore been proposed in a number of studies. Some authors have attempted to calculate the required quantity of coupling agent based on the number of active sites on the surface of the nanofiller that is available for chemical bonding [9], while others have used the size of the nanoparticles and the thickness of a monolayer of coupling agent [7] to evaluate the required volume of reagent. Another approach has involved calculations based on the surface area of the filling phase and the specific wetting surface of the silane coupling agent [10]. Despite this, there have been few detailed studies in which the degree of functionalization has been treated as a variable parameter in studies of nanocomposite systems [11,12].

This study set out with the objective of systematically varying the functionalization conditions of nanosilica (SiO_2_), with the aim of determining its effect on the structure and dielectric properties of a series of epoxy-based nanodielectrics. Specifically, the intention of the work was to test the generally accepted notion that, in nanodielectrics, surface interactions determine nanoparticle dispersion and that nanoparticle dispersion determines macroscopic properties through the formation of consequent interphases. Two functionalization methodologies have been considered, which involved hydrous and anhydrous conditions; only the former is reported here.

## 2. Materials and Methods

### 2.1. Materials

The epoxy resin used in this study was a diglycidyl ether of bisphenol A with an epoxide equivalent molar mass of 172–176 g·mol^−1^; D.E.R.™ 332, obtained from Sigma Aldrich, St. Louis, MO, USA. This was cured using a polyetheramine curing agent with an amine hydrogen equivalent molar mass of 60 g·mol^−1^; Jeffamine D-230, obtained from Huntsman Corporation, The Woodlands, TX, USA. The nanosilica was purchased from Sigma Aldrich and is characterized by a quote filler size of 10–20 nm; this was pre-treated with (3-glycidyloxypropyl)trimethoxysilane (GLYMO—Z-6040 from Dow Corning, Auburn, MI, USA) using the procedure described below.

Samples of nanosilica were modified to varying degrees by treating the nanoparticles with different quantities of coupling agent. Simplistic calculations suggest that, to functionalize 100 mg of spherical nanosilica particles 10–20 nm in diameter with a monolayer of GLYMO, ~20 mg of the coupling agent would be required; that is, a ratio of 5 parts nanosilica to 1 part GLYMO by mass. However, due to the uncertainties inherent in this calculation, seven samples were prepared using various ratios of nanosilica to GLYMO that span the above estimate (see Table 1). For this, the appropriate quantity of coupling agent was added to a pre-sonicated suspension composed of 800 mg of nanosilica in 25 mL of methanol and shaken vigorously. To inhibit GLYMO self-condensation reactions, the above reaction was conducted under acidic conditions; acetic acid was added according to the procedure specified by Ash *et al.* [13]. After 46 h, the nanosilica was recovered by first centrifuging and then washing with fresh methanol; this procedure was repeated five times to ensure that all excess coupling agent and all acid residues were removed from the system. Finally, the nanosilica was dried thoroughly at 40 °C under vacuum, to remove any residual solvent.

### 2.2. Nanocomposite Preparation

Nanocomposite samples were prepared from the above starting materials using the following procedure. First, 150 mg of the required nanosilica was introduced, with stirring, to 5.58 g of the D.E.R.™ 332 and sonicated for 1 h using a UP200s probe sonicator, Hielscher Ultrasonics, Teltow, Germany (110 W; 55% amplitude; pulsed mode with 0.7 s on, 0.3 s off). In order to prevent any excessive increase in temperature, the specimen was placed in a water bath throughout the sonication process and the water was replaced every 15 min. After sonication was complete, 1.92 g of hardener was added, as required to produce the optimum stoichiometric ratio of 1000:344 (epoxy:hardener), and the resulting mixture was thoroughly stirred with a magnetic stirrer for 15 min before being degassed under dynamic vacuum for 15 min. The resulting resin was then poured into a steel mold (pre-heated to 50 °C) containing a spacer, nominally 70 μm in thickness; all mold components were lightly coated with QZ-13 release agent, Huntsman Corporation, to aid removal of the resulting film specimen. All samples were cured at 100 °C for 4 h before, finally, being dried under vacuum at 40 °C for two weeks prior to testing.

### 2.3. Material Characterisation

To evaluate the effect of the surface functionalization treatment described above, specimens of the as-supplied nanosilica (*i.e.*, nanosilica NSU), NS0, NS4 and NS20 were subjected to combustion/CHN analysis (MEDAC Ltd., Woking, UK.), which, in this context, provides an indicative measure of the carbon content of each material. In addition, FTIR spectra were obtained from all nanosilicas, using a Thermo Nicolet 380 FTIR spectrometer (Thermo Scientific, Waltham, MA, USA) fitted with a Golden Gate single reflection diamond attenuated total reflectance (ATR) attachment. Spectra were collected with the spectrometer set in reflectance mode for wavenumbers ranging from 4000 cm^−1^ to 400 cm^−1^, using 32 scans at a resolution of 4 cm^−1^. All spectra were finally normalized with respect to the peak at 1100 cm^−1^, which, being related to the silica phase, provides a convenient reference.

The dispersion state of the nanofiller within each nanocomposite was examined using a field emission gun scanning electron microscope (FEG-SEM) 6500F SEM (JEOL Ltd., Tokyo, Japan). For this, film samples prepared as above were immersed in liquid nitrogen before being fractured to expose an internal surface. Images were obtained from gold sputter-coated specimens using an accelerating voltage of 15 kV and a working distance of 10 mm.

### 2.4. Nanocomposite Properties

AC electrical breakdown tests were conducted at room temperature on nominally 70 μm thick film specimens, in line with the ASTM D149 standard. Twenty random sites on each nanocomposite film were sequentially positioned between two 6.3 mm spherical ball bearing electrodes, whilst immersed in silicone oil and subjected to a 50 Hz AC ramp voltage that was increased at a rate of 50 V·s^−1^ (RMS) until failure. The ball bearing electrodes were routinely replaced after eight tests, to eliminate the possibility of surface pitting affecting the derived data. The sample thickness at each breakdown site was subsequently measured, such that the associated field at breakdown could be determined from the breakdown voltage. The resulting AC breakdown field data were processed using Weibull 7++ software (Reliasoft Corporation, Tucson, AZ, USA), assuming a two parameter Weibull distribution. The Weibull scale parameter, *α*, and shape parameter, *β*, were determined for each data set, together with 90% maximum likelihood confidence bounds.

The glass transition of each specimen was evaluated using a Perkin Elmer, Waltham, MA, USA, DSC 7 differential scanning calorimeter (DSC), after calibration with high purity indium. Samples weighing 5.0 ± 0.5 mg were enclosed in aluminum cans and held at 50 °C for 1 min, before the specimen temperature was raised from 50 to 120 °C at a rate of 10 °C·min^−1^ and then reduced rapidly to a nominal 30 °C. This procedure was executed twice in succession to erase the thermal history of the sample. Data for baseline correction were acquired from an unfilled DSC can and subtracted from the sample material second scan. The resulting baseline corrected data were finally normalized with respect to sample mass and the glass transition temperature, *T*_g_, of each nanocomposite was determined by taking the mid-point of the step-change in the DSC heat flow data. The associated change in specific heat capacity, Δ*C*_p_, and the width of the glass transition, Δ*T*_g_, were also routinely evaluated.

The dielectric response of the various epoxy systems was recorded using a Schlumberger, Houston, TX, USA, SI 1260 impedance/phase gain analyzer, in conjunction with a Solatron, Farnborough, UK, 1296 dielectric interface and a purpose built test cell with heating capability. Samples were prepared by sputter coating opposing circular gold electrodes measuring 32 mm in diameter onto each surface of the epoxy film. Dielectric spectra were obtained from each sample upon the application of a sinusoidal AC voltage of amplitude 1 V. Data were obtained at 20 °C intervals from 20 to 100 °C, over the frequency range 0.1 Hz to 0.1 MHz.

## 3. Results

### 3.1. Nanosilica Functionalization

**Figure 1:**
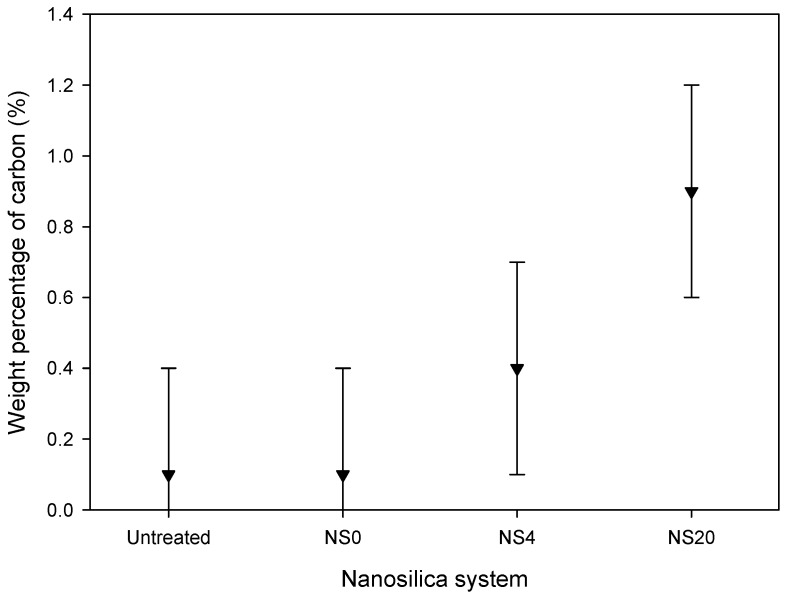
Combustion analysis data indicating an increase in nanosilica carbon content with increasing degree of surface modification. The error bars correspond to the accuracy of ±0.30% absolute quoted by MEDAC Ltd., Woking, UK.

The objective of surface treatment with an organosilane is to provide an amphiphilic bridge to promote desirable interactions between the filler and the surrounding matrix. The formulation used to produce each of the nanosilicas is listed in Table 1; each nanocomposite contained 2 wt % of the indicated nanosilica. The hydrolysis of the methoxy functional groups on the organosilane results in the formation of organosilane moieties with reactive silanol groups that can: (a) undergo self-condensation; or (b) react with hydroxyl groups on the surface of the nanosilica. Hydrogen bonds formed at the filler surface are replaced by covalent bonds upon drying, resulting in the functionalization of the nanofiller [7,14]. Figure 1 compares the weight percentages of carbon in untreated and treated samples of nanosilica. These data indicate that the carbon content in NSU and NS0 is equivalent; neither has been processed with GLYMO. This result also indicates that exposure to the acidic methanolic solvent does not lead to the adsorption of appreciable organic residues. The increasing levels of carbon detected in NS4 and NS20, although within the measurement uncertainties, are suggestive of successful surface modification.

**Figure 2:**
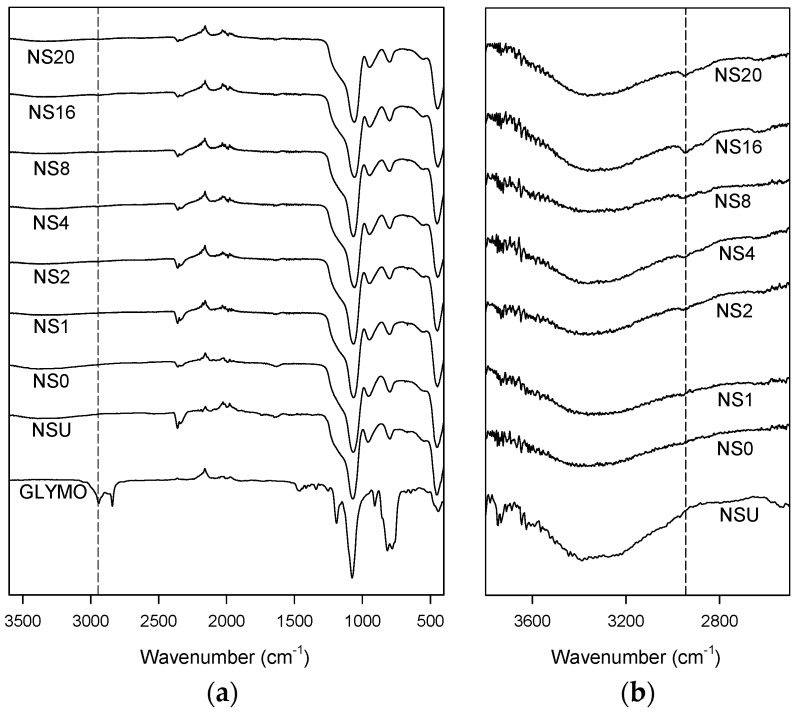
FTIR spectra obtained from all the nanosilica systems used in this study; the vertical dashed line indicates 2944 cm^−1^. (**a**) full spectral range; (**b**) detail showing the hydroxyl region.

Figure 2 contains Fourier transform infrared (FTIR) spectra obtained from all the nanocomposite systems listed in Table 1. All spectra show absorption bands from 1200 to 1000 cm^−1^, at 805 cm^−1^ and at 471 cm^−1^, which are characteristic of asymmetric Si–O–Si stretching, deformation of SiO_4_ tetrahedra and O–Si–O deformation respectively [15]. Data acquired from the GLYMO contain a strong absorption peak at 1250 cm^−1^, which is a consequence of the presence of the terminal epoxide ring, while the absorption in the range 2850 to 3000 cm^−1^ is representative of the CH_2_ groups that are associated with the GLYMO organic backbone [12,16]. Despite the lack of clear epoxide features in the spectra obtained from the surface-modified nanosilicas, there are observable differences in the region 3000 to 3700 cm^−1^, which is primarily associated with hydroxyl groups. As NSU and NS0 have not been chemically modified, they can be considered chemically equivalent as far as the GLYMO is concerned. Nevertheless, the strength of the hydroxyl band is rather less in the NS0 specimen; a decrease in the hydroxyl band is observed in processed silicas and is attributable to the removal of moisture from the surface during vacuum drying. There is also evidence of a minor additional peak at 2944 cm^−1^ for all the samples that had been functionalized with the GLYMO, as highlighted in Figure 2b, which increases in intensity with the volume of GLYMO used in processing. This feature correlates with the GLYMO CH_2_ absorption band indicated by the dotted lines in Figure 2 and therefore provides further evidence in support of the successful functionalization of the nanosilica.

Published accounts of organosilane chemistry describe the chemical pathways in terms of hydrolysis and condensation [14,17], before hydrogen bonds are formed with the surface of the substrate. However, such an idealized outcome constitutes an unlikely extreme, with other conformations being possible. In such cases, unreacted hydroxyl (silanol) groups will be retained within a disordered organosilica surface layer and, consequently, even in the case of NS20, where it would seem that a vast excess of GLYMO has been used, residual hydroxyl groups still exist near the particle surface, as evinced by Figure 2.

### 3.2. Nanocomposite Morphology

**Figure 3:**
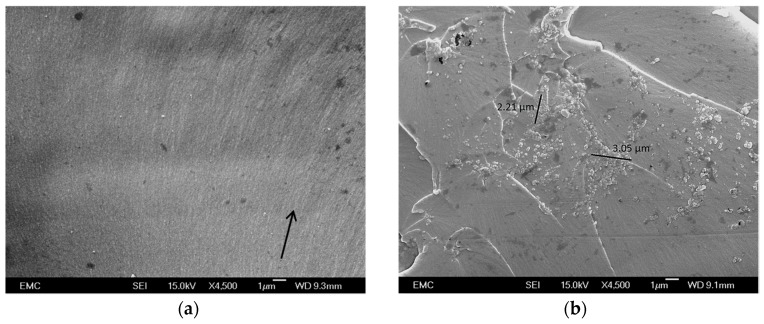

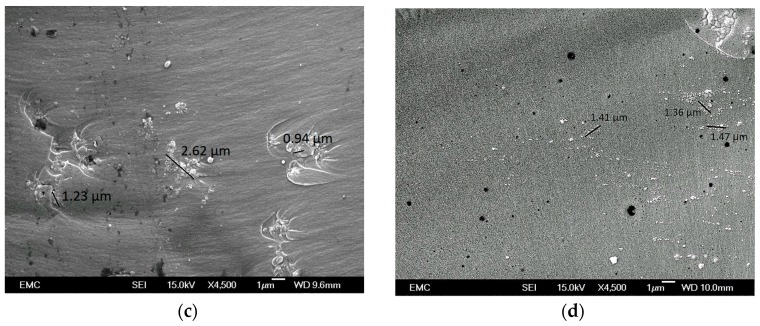
SEM micrographs showing representative fracture surfaces through a range of the materials considered here: (**a**) the unfilled epoxy specimen (EPX); (**b**) NC0; (**c**) NC4; and (**d**) NC16.

Although it is generally accepted that appropriate functionalization of nanoparticles leads to enhanced dispersion, we are not aware of any previous attempts to investigate how varying the degree of functionalization affects this. Figure 3a contains a typical scanning electron microscope (SEM) image of a cryofracture surface in an unfilled epoxy, which reveals a smooth texture in accordance with its amorphous structure. This image is in line with published work [18]. The surface striations indicated by the arrow are fractography features and lie orthogonal to the local crack propagation direction. In comparison, all nanocomposites were found to be characterized by much rougher surface textures that, locally, provide evidence of significant degrees of agglomeration, as shown in Figure 3b–d. Figure 3b shows an agglomerated region in sample NC0 that contains, in addition, bow-like fractography features associated with crack pinning [19]. Increasing the quantity of GLYMO used in functionalization results in a less agglomerated structure and in rather finer matrix surface textures.

While the presence of agglomerates in nanocomposites is not uncommon [20], the overall quality of the specimens shown above are markedly inferior to those reported by Nguyen *et al.* [18] in a recent study of epoxy/silica nanocomposites. In the work of Nguyen *et al.* [18], the nanosilica was introduced using a proprietary masterbatch system, Nanopox E 470 (supplied by Nanoresins), where the nanosilica is described as consisting of an agglomerate-free colloidal dispersion of surface-modified synthetic SiO_2_ with an average particle diameter of 20 nm. It is evident from the study of Nguyen *et al.* [18] that it is possible to produce agglomerate-free materials through the use of systems such as Nanopox E 470, albeit that this comes at the cost of neither knowing the nanofiller surface chemistry nor being able to modify it as required.

### 3.3. Dielectric Breakdown Strength

Table 2 contains breakdown parameters obtained from all the samples considered here, assuming two parameter Weibull statistics. From this, the breakdown strength, *E*_b_, of the unfilled resin, which in line with convention we equate to the Weibull α parameter, is given by 182 ± 5 kV·mm^−1^. This constitutes a reference point for all the other systems. Adding nanosilica directly without surface modification or solvent processing results in a decrease in breakdown strength to 173 ± 6 kV·mm^−1^. Reductions in breakdown strength on the inclusion of a nanofiller have been seen in many studies and have been ascribed to a number of effects, notably nanoparticle agglomeration [21,22]. Alternatively, a reduction in breakdown strength may stem from the existence of adsorbed moisture on the filler used to formulate this system (*i.e.*, NSU), as deduced from the infrared data. The presence of hydroxyl groups on unfunctionalized nanosilica will promote adsorption of water molecules, which has previously been linked to reduced breakdown strength [23,24] and described by Zou’s water model [25]. Indeed, the breakdown strength of the system containing the NC0 nanosilica, which has been subjected to both sonication and thorough drying, is increased by some 50 kV·mm^−1^ compared with NSU, where neither process was used. Increasing the mass of GLYMO used in the functionalization process results in a further increase in breakdown strength; the highest *α* value of 268 ± 12 kV·mm^−1^ being obtained from the system containing 2 wt % of NC8.

In view of the effect on breakdown strength of varying the chemical treatment and, by implication, nanoparticle/matrix interactions and resulting interphase regions [26,27,28,29], the effect of varying the nanoparticle surface chemistry on molecular dynamics [27,28,29] was examined thermally (*i.e.*, through DSC) and electrically (*i.e.*, through dielectric spectroscopy).

### 3.4. The Glass Transition

**Figure 4:**
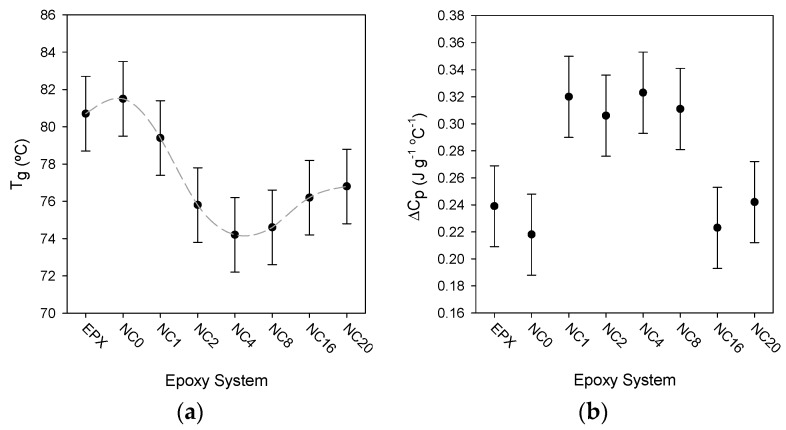
Effect of nanosilica surface chemistry on the observed DSC glass transition: (**a**) variation in the glass transition temperature with material system; and (**b**) variation in the change in the heat capacity, Δ*C*_p_, across the glass transition with material system.

All of the samples considered in this study exhibit a classical DSC glass transition, namely a singular step-change in heat capacity. The width of the transition, Δ*T*_g_, did not vary systematically with material formulation and always fell in the range 13.3 ± 1.5 °C; comparable results have been reported elsewhere [18]. Figure 4a shows the effect on *T*_g_ of varying the surface chemistry of the nanosilica. From this it is evident that introducing the unfunctionalized nanofiller (NC0) results in a small increase in *T*_g_ (0.8 °C), compared with the unfilled epoxy (EPX). Although this variation is less than the uncertainty in these data, a similar increase was reported by Couderc *et al.* [30], who observed a 0.5 °C increase in *T*_g_ for the same epoxy system on adding 2.5 wt % of nanosilica. For samples containing functionalized nanosilicas, the mean value of *T*_g_ initially decreases before increasing slightly again. Comparing EPX (*T*_g_ = 80.7 ± 2 °C) with NC4 (*T*_g_ = 74.2 ± 2 °C), for example, the difference in *T*_g_ between these two systems exceeds the uncertainties in the measurements and therefore, although the error bars overlap when considering any adjacent pair of points in Figure 4a, we suggest that the general trend indicated by the dashed line is real. To test this assertion, the change in the heat capacity, Δ*C*_p_, across the glass transition was determined for all samples and the effect of surface functionalization on this quantity is shown in Figure 4b. Where *T*_g_ is high in Figure 4a, generally, Δ*C*_p_ is low in Figure 4b; this anti-correlation is consistent with effects seen elsewhere in comparable systems [18], suggesting that the associated variations in *T*_g_ are indeed significant. Significant variations in the parameter Δ*C*_p_ have been seen in polyurethane-based nanocomposites containing organically modified montmorillonite (MMT), where a monotonic reduction in Δ*C*_p_ was seen as the volume of MMT in the system increased (0–5.7 vol %) [31]. This was interpreted in terms of an increasing rigid amorphous fraction, where reduced segmental mobility occurs as a consequence of immobilization within MMT tactoids. A comparable analysis is not possible here, since Figure 4b shows that Δ*C*_p_ both increased and decreased relative to the case of the unfilled system. Previously, it has been suggested that suitably modifying the surface chemistry of nanoparticles will restrict the overall mobility of the polymer chains within a nanocomposite, thereby increasing *T*_g_ [32,33,34]; in the case of nanocomposites containing graphitic nanofillers, the observed marked increase in *T*_g_ has been associated with a catalytic action of the nanofiller on the epoxy crosslinking reaction [35]. Elsewhere, it has been claimed that a reduction in *T*_g_ may be a consequence of poor interactions between the two phases [36]. However, the effects seen in Figure 4 are difficult to rationalize in either way. In the case of anhydride cured resins it has been demonstrated [18] that a change in the stoichiometric ratio of epoxy: anhydride results in a systematic variation in *T*_g_ and, since the various nanosilica systems discussed above may be expected to differ with respect to the number of epoxide groups adjacent to the surface, it is pertinent to consider the extent to which this may influence the overall stoichiometry of the cure reaction. Even taking the extreme case of NS20 and, unreasonably, assuming that all of the added GLYMO became bonded to the nanoparticles, this would only equate to an increase in epoxide content of ~3 mol %. The variations in *T*_g_ seen above cannot therefore be explained by gross stoichiometry effects and the invariance in the width of the glass transition indicates that no extensive interphase regions exist where local interactions affect main-chain dynamics.

### 3.5. Dielectric Spectroscopy

**Figure 5:**
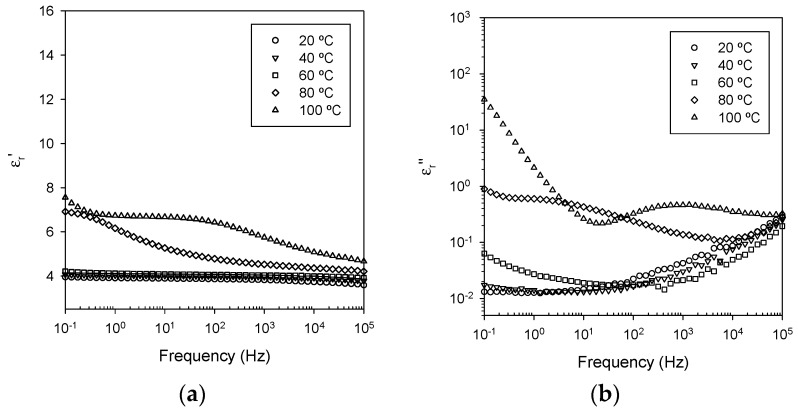
Dielectric data acquired from the unfilled epoxy (EPX): (**a**) real part of the relative permittivity; and (**b**) imaginary part of the relative permittivity.

Dielectric spectroscopy constitutes a powerful means of probing molecular dynamics in systems such as the nanocomposite samples considered here. Figure 5 presents data obtained from EPX, which reveal features that are typical of an unfilled epoxy [7,9,30]. At temperatures below *T*_g_, a slight reduction in the real part of the relative permittivity, ε_r_’, can be seen to occur in the frequency range 10^4^–10^5^ Hz. This is associated with the β relaxation, which has been ascribed to the rotation of the hydroxyether groups [CH_2_CH(OH)CH_2_O] in the backbone that results from crosslinking reactions between the epoxy and the hardener [37]. Being associated with segmental mobility, the β relaxation has been shown to be related to the mechanical yield behavior of network systems [38]. Data collected from the unfilled EPX at 80 °C and above are significantly different due to the glass transition which, in the DSC, is observed at ~80 °C. In the data acquired at 100 °C, the α relaxation is clearly visible in both the real and imaginary parts of the relative permittivity in the region 100–1000 Hz. Below this frequency range, the imaginary part of the relative permittivity, ε_r_*’’*, increases with decreasing frequency and, since (dlogε_r_*''*)/(dlogω) ≈ −1, we primarily associate this with a conduction process, presumably, as a consequence of the presence of residual chloride ions in the D.E.R.™ 332 epoxy resin [39]. However, in addition, there is an observable increase in the real part of the relative permittivity below 0.2 Hz, which may be associated with some degree of interfacial polarization, as a consequence of the accumulation of charge carriers at the electrodes [40,41].

**Figure 6:**
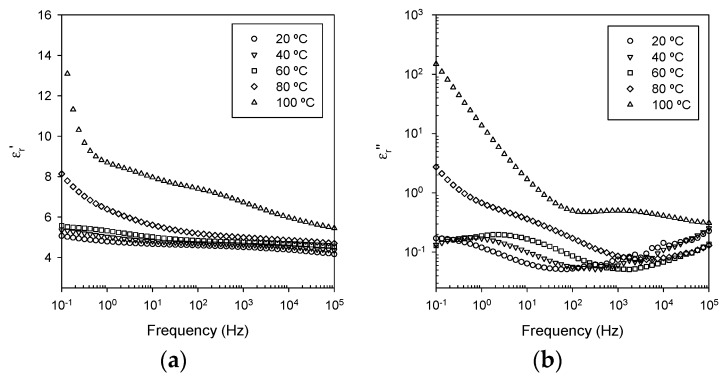
Dielectric data acquired from NC0, containing untreated nanosilica (NS0): (**a**) real part of the relative permittivity; and (**b**) imaginary part of the relative permittivity.

Figure 6 shows equivalent dielectric data obtained from NC0, from which it is evident that the introduction of 2 wt % of nanofiller has a marked effect on the dielectric response. At temperatures below the DSC *T*_g_, the β relaxation is still evident, particularly in the 20 °C trace but, otherwise, the value of the real permittivity is increased at all frequencies and at all temperatures. This effect is particularly evident below 10 Hz. Although such effects are not uncommon when adding an inorganic filler to a polymer, in the case of a system based upon 2 wt % of silica (silica real relative permittivity ~3.9 [42]) in an epoxy (epoxy real relative permittivity ~4.0 from Figure 5a), simple effective medium approaches would not predict such a result. As such, this increase in ε_r_*’* would appear to be a consequence of the presence of nanoparticle/matrix interfaces. At temperatures below *T*_g_, (*T*_g_ = 82 °C in this system from DSC) the imaginary permittivity data contain a relaxation peak that moves from ~10^−1^ Hz at 20 °C to ~10 Hz at 60 °C; since no equivalent feature can be seen in Figure 5b, we suggest that this is again a direct consequence of introducing the nanoparticles. Indeed, Couderc *et al.* [30] have described an α’ relaxation that they associated with adsorbed water molecules at the interface between the silica and the epoxy, which is also consistent with the increase in the magnitude of ε_r_*’* described above. Although NC0 was thoroughly dried prior to these measurements, this suggests that some residual bound water remains. At temperatures above the DSC *T*_g_, the additional internal boundaries between the nanofiller and the matrix lead to a pronounced low frequency polarization effect, which manifests itself as a steep increase in the real part of the relative permittivity below ~1 Hz at 100 °C. As in the case of the unfilled sample, the imaginary part of the relative permittivity increases markedly once the temperature approaches *T*_g_ and, at 100 °C, the low frequency variation of this quantity is close to (dlogε_r_*''*)/(dlogω) = −1, indicating that conduction processes within the epoxy matrix are again important.

In summary, the data discussed above can be interpreted in terms of three major processes, in addition to the glass transition:

1. Ionic conduction, which becomes significant in all systems at temperatures above the DSC *T*_g_.
2. Nanoparticle/matrix interfacial relaxation effects, that: (a) manifest themselves as an α’ relaxation peak in the imaginary permittivity; and (b) underlie the overall increase in ε*_r_’* seen in the nanocomposite.
3. Maxwell Wagner low frequency interfacial polarization at temperatures above the DSC *T*_g_, which appears as a marked increase in the real part of the permittivity at frequencies <1 Hz at high temperatures (*i.e.*, 100 °C in this work).

**Figure 7:**
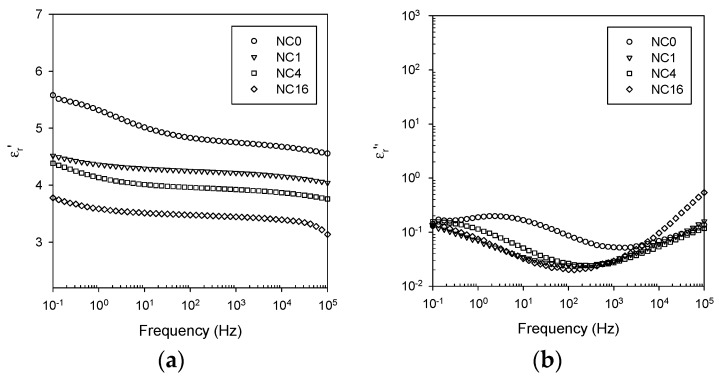
Dielectric data obtained at 60 °C comparing the response of nanocomposites NC0, NC1, NC4 and NC16: (**a**) real part of the relative permittivity; and (**b**) imaginary part of the relative permittivity.

**Figure 8:**
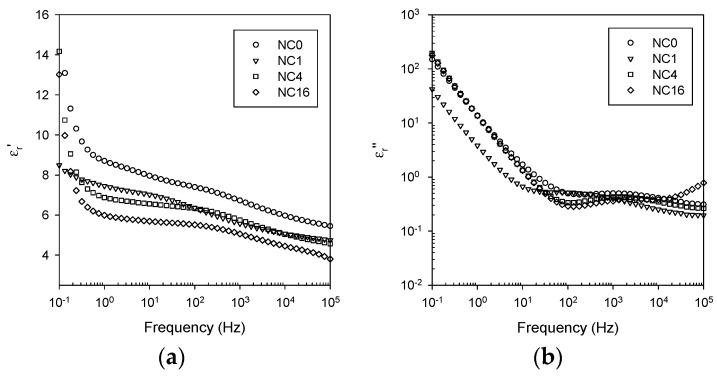
Dielectric data obtained at 100 °C comparing the response of nanocomposites NC0, NC1, NC4 and NC16: (**a**) real part of the relative permittivity; and (**b**) imaginary part of the relative permittivity.

To demonstrate the effect of surface chemistry on these features, spectra acquired from four different material formulations are compared, for the sake of brevity, at just 60 °C (*i.e.*, ~20 °C below the DSC *T*_g_) and at 100 °C (*i.e.*, ~20 °C above the DSC *T*_g_) in Figure 7 and Figure 8 respectively. From Figure 7, it is evident that below the DSC *T*_g_, surface functionalization has a pronounced effect on both the real and imaginary parts of the relative permittivity. In the case of ε_r_*’* (see Figure 7a), increasing the degree of functionalization progressively reduces this parameter until, in the case of NC16, its value is below that of the unfilled epoxy. The effect of filler loading level on the permittivity of nanocomposites has been considered by many workers and, in some cases, it has been reported that the addition of a low volume fraction of a high permittivity nanofiller can result in a reduction in the real relative permittivity [43,44]. While it is commonly proposed that the origin of this effect is related in some way to the existence of nanoparticle/matrix interactions, this assertion has not been fully justified. In this case, a supplementary factor can also be introduced, namely the availability of polar interfacial sites that are available for hydrogen bonding with water. If the overall increase in ε_r_*’* and the α’ relaxation are indeed associated with adsorbed water molecules at the epoxy/silica interface then, presumably, reacting the hydroxyl groups on NC0 with increasing quantities of silane will reduce the sites available to bind water, in addition to modifying the local molecular structure. Indeed, from the imaginary relative permittivity data shown in Figure 7b, it is evident that the α’ relaxation is much less apparent in all the GLYMO-treated systems, which reinforces this general concept. To conclude, the data shown in Figure 7 demonstrate that the overall dielectric response of a nanocomposite is determined not just by the components and the composition, but also by the nanoparticle surface chemistry and the consequent way in which the components interact. Specifically, the degree of polar character seems to be of considerable importance and not just in terms of matrix compatibility.

Figure 8 shows dielectric data acquired at 100 °C from the same samples previously shown in Figure 7. The spectra recorded at 100 °C are, however, significantly different from those collected at 60 °C, being dominated by conductivity and polarization effects, particularly at frequencies below 10^2^ Hz. Nevertheless, the magnitude of the real part of the relative permittivity again falls progressively with increasing surface functionalization, such that ε_r_*’* is again less in NC16 than in the unfilled EPX.

To permit further analysis, all data supporting this study are openly available from the University of Southampton repository at http://dx.doi.org/10.5258/SOTON/385542.

## 4. Discussion

A key paradigm that underpins the nanodielectric concept is that macroscopic properties are strongly affected by the presence of interphase regions, since: (a) the interphase constitutes a significant volume fraction of the system as a result of the high specific surface area of nanoparticles; and (b) that this fraction of the system is characterized by properties that are different from the bulk—perturbed molecular dynamics is commonly proposed [27,28,29]. The dielectric results presented above provide evidence of clear differences between the unfilled epoxy resin and those materials that contain nanosilica, which we ascribe to interfacial effects, in line with published work. Notably, the nanofilled systems all exhibit an α*’*-relaxation, which is strongly affected by the GLYMO treatment. While the DSC data demonstrate that the addition of nanosilica affects the glass transition and that the nature of this is dependent upon the nanofiller surface chemistry, the invariance of the width of the glass transition implies that the addition of nanoparticles does not, however, result in the development of a distinct interphase that differs significantly from the unperturbed matrix in terms of main-chain dynamics. In the case of controlled pore glasses, where interfacial interactions and confinement effects have been studied in detail, complex forms of behavior have been reported, which include two glass transitions, one below and one above the *T*_g_ measured for the equivalent bulk system [31] and a retardation and broadening of the glass transition [45]. In nanofilled polymers, Tsagaropoulos and Eisenberg [46] and Arrighi [47], for example, have both reported the existence of twin glass transitions, one corresponding to that of the unperturbed polymer and one related to polymer chains where interactions with the nanofiller result in reduced mobility. To rationalize the dielectric and DSC data, we suggest that the addition of the nanosilica affects the epoxy matrix in two ways, which can broadly be thought of in terms of the local behavior of small dipolar species and gross main-chain dynamics. For example, the data shown in Figure 7a equate to a progressive reduction in the polarizability of the system in going from NC0 to NC16 at a temperature below the DSC *T*_g_, where variations in the α’ relaxation are also apparent. We ascribe both of these effects to polar species at nanoparticle interfaces. Conversely, the glass transition, as revealed by DSC, implies that the whole of the matrix is being affected somewhat by the addition of the nanoparticles; this global effect does not, however, markedly influence the polarizability of the system (*i.e.*, ε_r_*’*) under conditions (temperature/frequency) where main chain mobility is negligible.

In many electrical applications, dielectric breakdown strength is an important technological parameter and, therefore, the effect of nanoparticles on this has attracted considerable attention. Andritsch *et al.* [48], for example, considered the effect of particle size on the breakdown strength of an epoxy resin containing hexagonal boron nitride and reported a monotonic increase in strength as the filler size was reduced at the constant loading level of 10 wt %. However, the effect of nano-structuring on breakdown is not always beneficial. In a recent study, the effect of surface chemistry on the AC breakdown strength of nanocomposites based upon nanosilica and a polyethylene blend has been described [49], in which this parameter was found to be independent of nanofiller loading level up to the point where nanofiller agglomeration became significant (5–10 wt % in the case of unfunctionalized nanosilica; >10% in the case of propyl-functionalized nanosilica), whereupon, inferior performance resulted. A similar dependence of breakdown strength on nanofiller loading level was also reported by Nguyen *et al.* [18] for epoxy/silica systems although, in this case, agglomeration of the nanofiller did not appear to be a significant factor. In the chemically treated and dried systems considered here, *E*_b_ increases for samples NC1 to NC8, before decreasing somewhat for more highly functionalized samples. Although changing the surface chemistry of the nanosilica does appear to have some effect on the dispersion state of the nanofiller, the SEM data indicate that this is relatively minor. That is, the pronounced improvement in breakdown strength that is evident in all functionalized systems is not related to improved dispersion. Indeed, in all the systems considered here, the nanoparticles are far from ideally dispersed and much more agglomerated than in the work of Nguyen *et al.* [18] where, despite the lack of agglomerated nanosilica, no significant increase in breakdown strength was seen.

The addition of epoxide functionality to a nanoparticle surface, as imposed here, may affect macroscopic properties in many ways. Covalent bonds will be formed with the curing resin, which will change the nature of the nanoparticle/matrix interface—mechanical integrity will be increased, for example [50]. In addition, electrically, the local density of states will change, thereby affecting charge transport through the system [41,51]. The process of functionalization additionally results in the replacement of hydroxyl surface groups with organic moieties, which can reduce adsorption of water molecules at nanoparticle surfaces [20]; adsorbed water has detrimental consequences. However, the silane will react with both the nanosilica surface and itself, such that residual silanol groups and epoxide functionality may be retained within the surface layer. It would appear that if this is too extensive, then the breakdown response becomes degraded.

In summary, the nature of the interactions that occur between nanoparticles and a matrix polymer are subtle and can manifest themselves in forms of behavior that can appear complex or even anomalous. In the study reported here, the consequence is an improvement in breakdown strength approaching 50% when the nanosilica was suitably functionalized. Since this cannot be related to nanoparticle dispersion effects and the DSC data provide no evidence of distinct interphase structures, we suggest that the origin of this improved performance is related to changes in interface characteristics and, in the absence of evidence for any major changes in the local structure, we suggest that variations in local trapping states/charge transport dynamics constitute a more likely explanation for the pronounced increase in breakdown strength reported.

## 5. Conclusions

A range of nanosilicas has been produced in which the surface chemistry has been systematically varied. The efficacy of the chosen organosilane chemistry has been demonstrated both by combustion analysis and FTIR spectroscopy. SEM examination of fracture surfaces indicates that the reaction of the nanosilica with increasing amounts of (3-glycidyloxypropyl)trimethoxysilane results in some improvement in nanoparticle dispersion, presumable, as a result of the presence of the introduced epoxy functionality. Nevertheless, in all cases, significant agglomerates remain and the systems described are, from a structural perspective, considerably less ideal than those that have been reported elsewhere [18]. However, despite this, significantly increased breakdown strength values have nevertheless been observed; this cannot be related to nanoparticle dispersion effects. In the absence of any direct evidence for distinct interphase regions between the nanosilica and the matrix polymer, which have been reported to result in multiple/broadened glass transitions, we suggest that the property enhancements we see stem from changes in the local chemistry, which result in changes in the local density of charge trapping sites and affect charge transport dynamics through the system. In particular, the substitution of hydroxyl surface character with organic moieties will markedly affect adsorption of water molecules onto nanoparticle surfaces, which manifests itself in the so-called dielectric α’ relaxation. However, the use of excess silane results in residual silanol groups (*i.e.*, polar groups) being incorporated into the organic-rich surface layer, which has negative consequences for macroscopic electrical properties, even if it leads to marginally improved nanoparticle dispersion.

## Figures and Tables

**Table 1:** Degree of nanosilica functionalization and designated codes for each sample. All nanocomposites contain 2 wt % of the appropriate nanosilica.

Nanocomposite	Nanosilica Filler	Ratio of Nanosilica to GLYMO
EPX	–	–
NCU	NSU	1:0
NC0	NS0	1:0
NC1	NS1	8:1
NC2	NS2	4:1
NC4	NS4	2:1
NC8	NS8	1:1
NC16	NS16	1:2
NC20	NS20	2:5

**Table 2:** Weibull parameters that characterize the breakdown behavior of the unfilled and filled epoxy systems.

Sample	*E*_b_ (kV·mm^−1^)	β
EPX	182 ± 5	14 ± 4
NCU	173 ± 6	13 ± 5
NC0	238 ± 10	9 ± 3
NC1	242 ± 7	13 ± 4
NC2	257 ± 19	5 ± 1
NC4	258 ± 11	9 ± 2
NC8	268 ± 12	8 ± 2
NC16	265 ± 16	6 ± 2
NC20	244 ± 13	7 ± 4

## Abbreviations

The following abbreviations are used in this manuscript:

GLYMO: (3-glycidyloxypropyl)trimethoxysilane
FTIR: Fourier transform infrared
SEM: scanning electron microscope
DSC: differential scanning calorimeter
